# Engaged genomic science produces better and fairer outcomes: an engagement framework for engaging and involving participants, patients and publics in genomics research and healthcare implementation

**DOI:** 10.12688/wellcomeopenres.17233.1

**Published:** 2021-11-15

**Authors:** Madeleine J. Murtagh, Mavis Machirori, Clara L. Gaff, Mwenza T. Blell, Jantina de Vries, Megan Doerr, Edward S. Dove, Audrey Duncanson, Jillian Hastings Ward, Rachele Hendricks-Sturrup, Calvin W. L. Ho, Amber Johns, Yann Joly, Kazuto Kato, Keiko Katsui, Judit Kumuthini, Fiona Maleady-Crowe, Anna Middleton, Richard Milne, Joel T. Minion, Mogomotsi Matshaba, Stephanie Mulrine, Christine Patch, Rosalyn Ryan, William Viney

**Affiliations:** 1 School of Social and Political Sciences, University of Glasgow, Glasgow, UK; 2 Ada Lovelace Institute, London, UK; 3 University of Melbourne, Melbourne, Australia; 4 Melbourne Genomics Health Alliance, Melbourne, Australia; 5 Newcastle University, UK, Newcastle Upon Tyne, UK; 6 University of Cape Town, Cape Town, South Africa; 7 Sage Bionetworks, Seattle, USA; 8 University of Edinburgh, Edinburgh, UK; 9 Wellcome, London, UK; 10 Participant Panel, Genomics England, London, UK; 11 Duke- Margolis Center for Health Policy, Washington, USA; 12 University of Hong Kong, Hong Kong, Hong Kong; 13 International Cancer Genome Consortium, Glasgow, UK; 14 Garvan Institute of Medical Research, Sydney, Australia; 15 Centre of Genomics and Policy, McGill University, Montreal, Canada; 16 GEM-Japan, Tokyo, Japan; 17 Osaka University, Suita, Japan; 18 Japan Agency for Medical Research and Development, Tokyo, Japan; 19 University of Western Cape, Cape Town, South Africa; 20 H3ABioNet/H3Africa, Cape Town, South Africa; 21 Genomics England, London, UK; 22 Wellcome Connecting Science, Cambridge, UK; 23 University of Calgary, Calgary, Canada; 24 Botswana-Baylor Children’s Clinical Centre of Excellence, Gabrorone, Botswana; 25 Northumbria University, Newcastle upon Tyne, UK; 26 Tibco.com, Palo Alto, USA; 27 Goldsmiths, University of London, London, UK

**Keywords:** Engagement and involvement; Genomics; Trust, Equality, Diversity and Inclusion; Fairness; Reciprocity; Collaboration; Global; Quality.

## Abstract

Genomic science is increasingly central to the provision of health care. Producing and applying robust genomics knowledge is a complex endeavour in which no single individual, profession, discipline or community holds all the answers.  Engagement and involvement of diverse stakeholders can support alignment of societal and scientific interests, understandings and perspectives and promises better science and fairer outcomes. In this context we argue for F.A.I.R.E.R. data and data use that is Findable, Accessible, Interoperable, Reproducible, *Equitable* and *Responsible. *Yet there is a paucity of international guidance on how to engage publics, patients and participants in genomics. To support meaningful and effective engagement and involvement we developed an *Engagement Framework for* *involving and engaging participants, patients and publics in genomics research and health* *implementation*.

The *Engagement Framework *is intended to support all those working in genomics research, medicine, and healthcare to deliberatively consider approaches to participant, patient and public engagement and involvement in their work. Through a series of questions, the *Engagement Framework* prompts new ways of thinking about the aims and purposes of engagement, and support reflection on the strengths, limitations, likely outcomes and impacts of choosing different approaches to engagement. To guide genomics activities, we describe four themes and associated questions for deliberative reflection: (i) fairness; (ii) context; (iii) heterogeneity, and (iv) recognising tensions and conflict.

The four key components in the *Engagement *provide a framework to assist those involved in genomics to reflect on decisions they make for their initiatives, including the strategies selected, the participant, patient and public stakeholders engaged, and the anticipated goals. *The Engagement Framework* is one step in an actively evolving process of building genomics research and implementation cultures which foster responsible leadership and are attentive to objectives which increase equality, diversity and inclusion in participation and outcomes.

## Disclaimer

The views expressed in this article are those of the author(s). Publication in Wellcome Open Research does not imply endorsement by Wellcome.

## Introduction

Genomic ^1^ science is increasingly central to the provision of health care. Producing and applying robust genomics knowledge is a complex endeavour in which no single individual, profession, discipline or community holds all the answers ( Murtagh *et al*., 2011). Aligning societal and scientific interests, understandings and perspectives of diverse stakeholders promises better science and fairer outcomes ( Murtagh *et al*., 2018; Nunn *et al*., 2019; Shabani *et al*., 2021). To achieve this requires responsible leadership which enables collaboration between those working in the area of genomic science and those who choose to contribute their genomic data, or who are impacted by the application of genomics: (1) participants of studies that inform scientific development in genomics, (2) patients who may be directly affected by genomic science and technologies, and (3) members of the public globally. This is best done by involving and engaging with those participants, patients, and publics ( Bruni *et al*., 2008; Cox *et al*., 2009; Greenhalgh *et al*., 2019; Luna Puerta *et al*., 2020; Murtagh *et al*., 2017; Ochieng *et al*., 2021; O'Mara-Eves *et al*., 2015). Engagement and involvement practices, done well, can enhance *trust*, support understanding of *diversity* and the *impact* of research, increase the *value*, *relevance* and *quality* of genomics and support science/society *collaboration*. Allowing for *continuity* and change *, e*ngagement and involvement can lead to better long-term insights to support societal alignment and respect for participants, patients and publics over the life course of genomics research and implementation. We consider *trust*, *diversity, impact, value*, *relevance* and *quality, collaboration and continuity* (See Box 1) the foundational logics of engagement and involvement practice. Engagement and involvement can foster greater fairness, justice and reciprocity in genomics. Yet there is a paucity of international guidance on how to engage publics, patients and participants in genomics ( Erikainen *et al*., 2020).

**Box 1: Foundational logics of engagement and involvement**

*Trust*: Researchers and clinicians depend on people contributing large amounts of personal data. While transparency in how personal data is used is important, transparency of how and to what extent participants, patients and publics will be involved, can support the building of trustworthy relationships - that is, it can lead to gaining the trust of people whose data is being investigated. Such relationships of trust may facilitate people providing their data, and so engagement can be a way to help ensure accountability and transparency in science especially, but not exclusively, where activities are publicly funded.

*Collaboration*: Genomics is a collective endeavour, bringing together professionals from many disciplines as well as participant, patient and public stakeholders from all walks of life. Improved collaboration across these different groups allows for the identification of new research and clinical priorities.

*Diversity*: Genomics research and clinical practices are most effective when they capture data from people across the widest possible demographic and geographic backgrounds.

*Impact*: Depending on the project’s design, genomics can reveal significant study results and information about individuals and their families. It can also tell us about unrelated people who are part of the same community. Not all such information is welcome, and stakeholders may not be affected by, or respond to, this information in the same way. Engagement will help to identify potential positive and negative impacts at both the individual and community level.

*Value*: Engagement with participants, patients and publics stakeholders on research and clinical care can leverage real-world evidence to maximise the utility and outputs of genomic information and practices. Engagement can help create additional value within projects through closer alignment of professional objectives to participant, patient and public interests and concerns. Such added value can in turn be made apparent through systematic evaluation of the participant, patient and public engagement and involvement process. What are the *Engagement Framework*’s key components?

*Relevance*: Participant, patient and public stakeholders have experience which gives them unique knowledge and expertise of the potential impact of genomics in their lives. They are well placed to help shape genomics research and transform clinical work. Involving them in genomics initiatives ensures that genomics research is relevant to those impacted by its findings.

*Quality*: Involving participant, patient and public stakeholders in research can improve not only the relevance and impact of genomics, but the quality and quantity of data collected. Better data means better science, which in turn enriches the experience of genomics for everyone involved.

*Continuity*: Genomic analysis produces new knowledge long after a genome has been collected and sequenced. Ongoing engagement with participant, patient and public stakeholders as knowledge is generated (after initial data collection and sequencing) is important to see whether the findings and the knowledge are acceptable. As new genomic technologies continue to emerge, the knowledge and technologies should be explored with different participant, patient and public stakeholders.

To support meaningful and effective engagement and involvement we developed a framework for involving and engaging participants, patients and publics in genomics research and health implementation (hereafter, *Engagement Framework*. The *Engagement Framework* was collaboratively developed with international, multidisciplinary, multi-professional and multi-community stakeholders and members of the Regulatory and Ethics Working Group and Global Alliance for Genomics and Health’s (GA4GH) community. The GA4GH is an international non-profit organisation which influences the work of over 600 health research organisations around the world. GA4GH supports equality, diversity and inclusion (EDI) groups focusing on the diversity of perspectives and expertise within GA4GH community, and on how products and standards can be designed or implemented in ways that progress EDI. The *Engagement Framework* draws upon knowledge and expertise on engagement from within the GA4GH community. The *Engagement Framework* is also informed by its Driver Projects (real-world genomic data initiatives that help guide the GA4GH’s development efforts and pilot its tools) and the Genomics in Health Implementation Forum (GHIF), the Your DNA Your Say study ( Middleton *et al*., 2020; Milne *et al*., 2021), and from other collaborators interested in genomics and engagement. Various engagement activities are already undertaken by driver projects and initiatives within the GHIF and Driver projects (available from GA4GH Secretariat) illustrate the breadth of engagement activities already being undertaken. The *Engagement Framework* is underpinned by principles of fairness, justice and reciprocity and builds on the mission of the GA4GH, its *Framework for Responsible Sharing of Genomic and Health-Related Data and commitment to Diversity and Inclusion.*

This *Engagement Framework* is for all who are interested in engaging with different stakeholders around genomics and data sharing and aims to encourage all those involved in genomics activities across and beyond GA4GH initiatives and projects to consider undertaking public engagement in their work.

## How to use the Engagement Framework

The *Engagement Framework* acknowledges the importance of deliberative reflection about the purposes and strategies of engagement as forming part of a project’s life-course. Such deliberation and reflection aims to recognise the importance of diverse demographic and geographic backgrounds in genomics engagement for achieving justice and fairness. Deliberative approaches also recognise the importance of evaluating engagement strategies to enhance learning and future work. Through a series of questions, the *Engagement* Framework prompts new ways of thinking about the aims and purposes of engagement, and reflection on the strengths, limitations and/or likely outcomes and impacts of various approaches to engagement. These questions explore how engagement and involvement can better reflect different participant, patient and public stakeholders’ demographics and geographies.

The *Engagement Framework* does not promote a singular understanding or approach to engagement. Nor does it rehearse the benefits and limits of particular forms of engagement and involvement as this is ably done elsewhere ( Aitken & Burley, 2021; Chuong & O’Doherty, 2021; Erikainen *et al*., 2021; Luna Puerta *et al*., 2020).

To guide genomics activities four themes are central to participant, patient, and public stakeholder engagement:

- Fairness
- Context
- Heterogeneity
- Recognising tensions and conflict

## Developing engagement through deliberative reflection

Here we outline the key considerations for deliberative reflection in developing engagement and involvement strategies. We provide a definition of each theme and propose questions to guide users of the *Engagement Framework* through the processes of thinking critically about engagement work and considering how to choose an approach best suited to their project. Although the themes are discussed individually, in practice each impacts the application of the others. As such, the themes are meant to be read as complementary rather than standalone.

### 1.  Fairness

Incorporating the perspectives and experiences of participant, patient and public stakeholder into genomic research and health implementation is essential for both scientific and ethical reasons. Fairness, in the context of genomics science, aims for the equitable distribution of genomics outcomes and benefit-sharing across diverse stakeholders or communities (public as well as professional), irrespective of geographic or demographic differences. Attention to fairness ensures that research and healthcare are undertaken responsibly and do not produce or exacerbate the inequity and inequalities people already face. While management of genomic data must now commonly adhere to F.A.I.R. principles (Findable, Accessible, Interoperable, Reproducible) ( Wilkinson *et al*., 2016), achieving F.A.I.R. data, does not necessarily result in fairness in data use ( Leonelli *et al*., 2021). Indeed, enabling greater data use for global genomics science may cause inequities and inequalities where data use or sharing is undertaken without fully accounting for diversity, inclusion and equality. Instead, we argue for F.A.I.R.E.R. data and data use that is Findable, Accessible, Interoperable, Reproducible, *Equitable* and *Responsible.*

The *Engagement Framework* therefore argues that *all* genomic activities must aim to be equitable, and researchers and healthcare professionals should be responsible to their communities. Aiming for equity and responsibility can produce *fairness* by encouraging listening to and incorporating diverse participant, patient, and public stakeholder voices ( Fricker, 2007; Kaye *et al*., 2018; Pratt & Hyder, 2016). Considering society means re-evaluating what being *fair* looks like from participant, patient, and public perspectives. This ensures that:

(1) impacts on people – individually or collectively – are considered;
(2) power differentials between and among professionals and non-professionals are considered and rebalanced where possible;
(3) different perspectives are considered in relation to who asks the questions and who answers them. For each of these points, how stakeholders might benefit or whether they have a say in those matters are important additional issues to consider; and
(4) sufficient consideration is given to the voices of vulnerable or marginalize peoples, and the unique challenges they face.

To this end, the CARE Principles for Indigenous data governance ( Carroll *et al*., 2021) are a good resource that can help projects think further about engagement and involvement.

Questions to enable fair engagement:

- What is the purpose of the activity I choose? What do I want to achieve?
  - What is the best way to achieve that purpose (strategies, methods/activities, research and practice)?
- How am I defining my stakeholder groups?
- Why do I want to engage this stakeholder group? Why choose them rather than another group of stakeholders?
- How inclusive is this stakeholder group?
  • What promises have been made to invite, engage and involve that audience? Are they achievable? How will the chosen stakeholders be involved in the project pathway?
  • When is the ideal time to involve different people?
  • What might different audiences think of the results of the project/activity?
  • Could the way a stakeholder group, research question/purpose or activity is defined cause or reproduce
    • misconceptions, stigma, oppression, etc.?
    • inequalities?

### 2.  Context

The context in which an activity occurs includes the range of circumstances under which research or health implementation takes place. Context reflects many different elements such as a genomic project’s stage of development, its connection to other projects, the genomic/health condition in question, where the project’s activities are conducted, and the potential project funding priorities.

Context also evolves as priorities and people change over time and this will drive decisions on who is involved in engagement. The particular contexts of engagement will shape the work by influencing what is needed, what is possible, what is acceptable and what is achievable. Good engagement practices will involve regularly adapting strategies to ensure that the purpose of engaging stakeholders aligns with the context being considered.

Questions to support context-sensitive engagement:

- What is the context of the initiative?
- In this context, whose perspective is being championed by the engagement activity?
  • Could a different perspective provide more benefit or value to everyone involved?
- Is the engagement activity in this context a sustainable solution?
  • As the project evolves, is the same engagement activity with the same stakeholders sufficient or does something need to change?
- Is the purpose of pursuing this specific engagement, in this context, enough to ensure fairness to all involved? Does it sufficiently account for or consider histories and cultures of oppression?

### 3.  Heterogeneity

Heterogeneity refers to the diversity and inclusivity among and within the groups of people involved in engagement activities related to a genomics project, which may themselves be varied. Like considerations of context, heterogeneity acknowledges that the nature and scope of engagement may need to be tailored to both differences and similarities among people within specific settings. A single project might use one or more engagement strategies at different times and with different people; these may be one off, continuous and/or co-productive. This way, engagement can involve diverse stakeholders or achieve different sets of outcomes.

Participant, patient, public stakeholders, and stakeholder organisations each bring unique experiences, knowledge and values to genomic science. It is useful for the person/team developing the engagement strategy to remember that none of these groups are the same, and not to expect the same outcomes out of engagement practices. Being open to potential differences between and within groups allows those undertaking engagement work to explore a diversity of potential engagement activities before deciding on specific strategies.

Questions to support heterogeneity-sensitive engagement:

- How can multiple perspectives be heard and incorporated into genomics initiatives?
- Are there potential perspectives not being heard?
  • What does diversity look like? Why do you want to pursue it?
- How can these differences shape the purpose of the engagement overall?
  • How can they shape the purpose of the engagement from the time a decision to undertake engagement is made?
- What decisions around engagement in the project have been taken before and what might need to be changed?
  • What will be the potential impact on people’s lives?

### 4.  Recognising tensions and conflict

Genomics initiatives can create both excitement and anxiety. These can lead to tensions, and, on occasion, disagreements between stakeholders. The central aim of engagement - to bring different perspectives together - can draw attention to differences in opinions, values, and beliefs which can be experienced as tensions or conflict.

Tensions, even disagreements, should not be seen as damaging or a sign of failure, but rather opportunities for dialogue, deliberation and understanding. When professionals are open to exploring why participant, patient and public stakeholders might be responding in a particular way, such openness can promote better understanding of what causes tensions and can promote fairness in how professionals respond. *All* stakeholders in genomics should be given opportunities to acknowledge and question one another without prejudice or penalty.

Supporting stakeholders to highlight areas of concern promotes transparency and can offer a way to address tensions. This may not be required (or appear to be required) at the start of a project or even as tensions emerge over time, but exploration of different viewpoints early in the engagement process can assist with management of tensions as they arise. Providing time to constructively uncover differences in experience, knowledge, and values encourages a transparent process and can avoid engagement becoming unproductive. While tensions can necessitate difficult conversations, when sensitively and carefully worked through these can support engagement and help stakeholders evaluate whether their concerns have been recognised and considered appropriately or not. By actively embracing tensions rather than glossing over them, engagement can avoid being reduced to merely a tick-box exercise.

Good practice includes project leaders taking responsibility for developing acceptable approaches to manage or resolve tensions. Understanding the experiences and potential worries of participants, patient, and public stakeholders allows genomic researchers and health care professionals to adapt their practices and work with their specific communities. While a robust and effective communication strategy is vital, uni-directional information flows will not support engagement or ameliorate tensions or conflict. Deliberative reflection and feedback are needed to create an effective dialogue. Collaborative or co-productive approaches to engagement can facilitate ongoing conversations between all stakeholders and thereby also work to ameliorate tensions. 

Good communication is fundamental for establishing respectful, sustainable relationships. Achieving good communication means allowing all stakeholders to explain their perspectives and/or positions, listening carefully and seeking to understand different views, and respectfully acknowledging differences. This approach to communication can be useful for resolving conflict in the pursuit of commonly acceptable and agreed ends. Deliberative reflection implies committing to listening with the intent to create collaborative action (if required), rather than listening for its own sake, and can lead to improved outcomes for all people involved. Additionally, project leaders should be willing to seek independent support, if needed, to facilitate discussion and/or resolve conflict, as well as change the direction of their projects in response to participant, patient, and public stakeholder concerns.

Engagement addressing tensions/conflict considers the following questions:

- What issues or practices might be a source or cause of tension?
  - Who is responsible for managing or responding to tension?
  - How could responses to people’s concerns or differences of opinion avoid blame, shame, or stigma, and instead, create better outcomes for all?
- How can the initiative support better engagement between stakeholders so that it fosters an open and trusting environment?
- How are activities, intentions and findings/outcomes being communicated in ways that encourage diverse feedback?
- How can different perspectives work together constructively?
- How are tensions and conflicts resolved and managed at different levels and times in the project?
  - How will differing or conflicting perspectives be accommodated and accounted for?
- How do all stakeholders show they are listening, acknowledge disagreements (including amongst themselves) and are responsive to different perspectives?

## Engaging effectively

Having considered the key components and questions above, the next steps when thinking about, or choosing, an engagement approach is to align the approaches to engagement with the purposes of that engagement: in research terms this is akin to choosing the right methods and methodology to answer a research question.

As part of the process of considering engagement approaches, we also recommend building in time and resources to evaluate those approaches, though we acknowledging more work is needed to guide how best to effectively evaluate engagement and involvement ( Nunn *et al*., 2019). In so doing, the value of engagement can be considered in view of the intended aims and outcomes of the project. It is desirable to evaluate engagement practices against their aims and outcome goals as an ongoing process both during and after the project. Outcomes themselves can additionally be evaluated against the aims and reasons of conducting engagement. Assessing outcomes of engagement activities can be done quantitatively or qualitatively ( Russell *et al*., 2020). The potential ways of conducting evaluations of engagement are not covered in the *Engagement Framework*, but models such as H3Africa-CEBioGen, ADRUK, MESH, STARDiT, and resources provided by others such as INVOLVE, Consumer and Community Involvement Program, Imperial College London's public involvement resource, National Institute for Health Research and the International Association for Public Participation, offer specific approaches for evaluating engagement that might be useful (See Box 2). Considering the strengths and limitations of each engagement activity (i.e., what it can and cannot do), will better equip those running such activities to evaluate whether their chosen approach was fit for purpose or achieved what was intended. Further, publishing such evaluation will enable others to design engagement strategies to better achieve their own aims.

**Box 2: Engagement resources**

GA4GH Framework for Responsible Sharing of Genomic and Health-related Data - https://www.ga4gh.org/genomic-data-toolkit/regulatory-ethics-toolkit/framework-for-responsible-sharing-of-genomic-and-health-related-data/

GA4GH Your DNA, Your Say (Participant Values Survey) https://www.ga4gh.org/news/your-dna-your-say-the-why-and-the-how/

GA4GH Consent Policy https://www.ga4gh.org/wp-content/uploads/GA4GH-Final-Revised-Consent-Policy_16Sept2019.pdf

Consumer and Community Involvement Program: https://cciprogram.org/researcher-services/types-of- community-involvement/

GIDA – Global Indigenous Data Alliance. Care Principles of Indigenous data governance: https://www.gida- global.org/care

H3Africa – CEBioGen. Developing best practices of community engagement for genomics and biobanking in Africa: https://h3africa.org/index.php/developing-best-practices-of-community-engagement-for-genomics- and-biobanking-in-africa-cebiogen/

International Association for Public Participation ( www.iap2.org): https://cdn.ymaws.com/www.iap2.org/resource/resmgr/pillars/Spectrum_8.5x11_Print.pdf

INVOLVE 2015: https://www.invo.org.uk/wp-content/uploads/2017/08/Values-Principles-framework- Jan2016.pdf

National Institute for Health Research (NIHR) School for Primary Care Research - https://www.spcr.nihr.ac.uk/PPI/what-is-patient-and-public-involvement-and-engagement

OmicsXchange Podcast, Episode 9 (27 August 2020) The importance of diverse perspectives in standards development: An interview with Laura Paglione. https://www.ga4gh.org/news/omicsxchange-podcast- episode-9-the-importance-of-diverse-perspectives-in-standards-development-an-interview-with-laura- paglione/

MESH community engagement network: https://mesh.tghn.org

Public involvement – Imperial College London Patient Experience Research Centre https://www.imperial.ac.uk/patient-experience-research-centre/ppi/

STARDIT: Standardised Data on Initiatives: Alpha Version. Nunn, J. S., Shafee, T., Chang, S., Stephens, R., Elliott, J., Oliver, S., … Orr, N. (2019, September 20). https://doi.org/10.31219/osf.io/5q47h

The Trust Project, Challenges and opportunities of community engagement. https://www.scidev.net/sub-saharan-africa/health/feature/community-cooperation-health-research-kemri.html

WHO & UNITAD Toolkit for research and development of paediatric antiretroviral drugs and formulations, *Module 6: Community engagement*. www.who.int/hiv/pub/6.pdf

The type and impact of engagement, the spectrum of strategies, and the level of interaction between different groups or people therefore forms a complex landscape which will influence the strategies and outcomes. Despite this complexity, the underlying principles of fairness, justice and reciprocity must remain of central importance.

Considering different approaches to engagement

The following questions can be useful when determining the strengths and limitations of the different engagement approach. These questions can guide decisions about which forms of engagement to use and can help ensure these align with the overall aims of the project:

- How will I assess the value and outcome of the strategy or engagement activity?
- Does the choice of engagement activity support the context?
- What are the limitations of the activity chosen - what can it do and what can it not do?
- Is the chosen activity appropriate to the purpose of the research and the resources available?
- Are the outcomes of the activity in line with the purpose of engagement? How will we know?
- How might the activities or outcomes have been different with or without participant, patient and public engagement and involvement?

## Conclusion

The four key components in the *Engagement Framework* – Fairness, Context, Heterogeneity and Recognising tensions and conflict – provide a framework to assist those involved in genomics to reflect on and consider the strengths and limitations of the engagement decisions they make for their initiatives. These can include the strategies selected, the participant, patient and public stakeholders chosen, and the anticipated outcomes. Trying to define fair outputs and participation in such situations are important topics that are, nevertheless, outside the scope of the *Engagement Framework*. However, as multiple stakeholders have diverse expectations, working through some of the many issues and questions we have raised, can help to take those views into account when designing appropriate engagement approaches. By considering the strengths and limitations of what may be achieved by different approaches to engagement, it becomes possible to evaluate whether engagement work is achieving its intended purpose, and to identify where it may be further improved.

The *Engagement Framework* is one step in an actively evolving process of building genomics research and implementation cultures which foster responsible leadership and are attentive to equality, diversity and inclusion. In the spirit of promoting and using diversity to enhance genomics initiatives, we encourage those involved in genomics to always be vigilant about deep seated social injustices and practices or arrangements that are inconsistent with the principles of fairness, justice and reciprocity and maintain a deliberative and reflexive orientation by coming back to the question:

- What would engagement and involvement look like if a different method had been used or a different group had been approached?

## Data availability

No data are associated with this article.

## Footnotes

We use the term ‘genomic and genomics’ as shorthand to refer to the range of ‘omics’ sciences, and medicine, healthcare implementation practices
