# Supplementary material for: Engaged genomic science produces better and fairer outcomes: an engagement framework for engaging and involving participants, patients and publics in genomics research and healthcare implementation
Source: Wellcome Open Res. 2021 Nov 15;6:311. [Version 1] doi: 10.12688/wellcomeopenres.17233.1 (PMC9086526; doi:10.12688/wellcomeopenres.17233.1)
Supplement: Peer review history

# Peer review: Reviewer response for version 1

**DOI:** 10.21956/wellcomeopenres.19047.r50245

**Published:** 2022-05-09

**Authors:** J. Lloyd Michener

**Affiliations:** 1 Department of Family Medicine and Community Health, Duke University School of Medicine, Durham, NC, USA

This Open Letter concisely makes the case for considering fairness, context, heterogeneity, and recognition of tension and conflict as part of the role of genetics researchers, and offers an “Engagement Framework for involving and engaging participants, patients, and publics in genomics research and health implementation.” The Framework was developed by an international collaborative under the Regulatory and Ethics Working Group and Global Alliance for Genomics and Health’s community, which comprises over 600 health research organizations globally, and builds on its *Framework for Responsible Sharing of Genomic and Health-Related Data,* and its commitment to Diversity and Inclusion. The Framework itself is an expansion of the F.A.I.R Principles (Findable, Accessible, Interoperative, Reproducible) for management of genetic data, and adds Equitable and Responsible to make F.A.I.R.E.R  data and data use. Importantly, while the authors state that the Framework was developed by multicommunity stakeholders, it is not clear whether members of community organizations were involved, outside the researcher community, and if so, to what degree.

 The Framework aligns quite closely with other, broader guidance for community engagement in research. The (US) federal guidance, “ Principles of Community Engagement, 2 ^nd^ edition” outlines nine principles which are substantially similar, but with a greater emphasis on the centrality of community autonomy and long-term commitment. This report may be worth adding to the resource list, especially as it reportedly has been viewed more than a million times. A third edition, not yet released, will reinforce these principles and add a tenth, on trustworthiness.

 A just-released and already widely viewed report from the (US) National Academies of Science, Engineering and Medicine, on methods of assessing authentic community engagement, similarly emphasizes the central role of communities, and of the importance of trust and culture, ongoing relationships, shared governance, and equity (among others).  A key observation is “ The fundamental question is not whether entities think they are engaging communities, but whether communities feel engaged.”. This too may be a useful resource.

 Interestingly, the NIH’s All of Us, which includes genetic research, also focuses on participants as partners who help shape the program, and the need for transparency and trust- including the need to return genetic results to participants, a topic not directly addressed in the Framework. It is not clear if such specificity around expected behavior by genetic researchers is a US anomaly, or the result of having community participants actively engaged in the design and implementation of research protocols.

 These recent reports and efforts underscore the importance of the Framework offered in this Letter and reinforce that participant and community engagement is an evolving process and that engaging community members as partners, not just as subjects, will help earn and maintain community trust in research (and research institutions). Since support of community and participant engagement is a broad concern across the research enterprise, having genomic researchers involved in larger institutional and national efforts in community engagement in research will help all researchers to be perceived as trustworthy, and will help ensure that the recommendations outlined here are part of larger efforts to increase engagement, equality, diversity and inclusion in research and in achieving health for all.

Does the article adequately reference differing views and opinions?

Yes

Are all factual statements correct, and are statements and arguments made adequately supported by citations?

Yes

Is the Open Letter written in accessible language?

Yes

Where applicable, are recommendations and next steps explained clearly for others to follow?

Yes

Is the rationale for the Open Letter provided in sufficient detail?

Yes

Reviewer Expertise:

Community engagement in research; multisector partnerships for health equity.

I confirm that I have read this submission and believe that I have an appropriate level of expertise to confirm that it is of an acceptable scientific standard.

# Peer review: Reviewer response for version 1

**DOI:** 10.21956/wellcomeopenres.19047.r48669

**Published:** 2022-04-06

**Authors:** Colin Mitchell

**Affiliations:** 1 University of Cambridge - PHG Foundation, Cambridge, UK

This open letter sets out a clear and valuable framework for genomics initiatives to use when considering the nature and form of engagement that is best suited to the purposes and context of the initiative. The authors (as part of the Global Alliance for Genomics and Health or GA4GH) identify a gap in international guidance for decision-makers who have recognised the importance of engagement for high quality science which fosters fairness, justice and reciprocity, on *how* to approach engagement in their initiative. The resulting framework, developed through international and multidisciplinary/multicommunity collaboration, encourages deliberative reflection across four themes that are central to engagement: fairness; context; heterogeneity; tensions and conflict. A set of questions under each theme are posed for users to reflect on. This is a user-friendly approach which should provide a strong starting point for those in the design stage of an initiative as well as (as the authors stress) for deliberative reflection during a project’s life-course.

 Importantly, this framework is neutral as to the precise engagement or participation methods that may be adopted. The intention is to provide a framework of general relevance that can lead to a decision about the most appropriate form(s) of engagement in the context of a particular initiative. Likewise, the framework does not cover models for evaluating engagement but with both these topics there is helpful signposting to relevant literature, exemplars and resources.

 In my view the framework (and this letter) successfully achieves its goals. It provides a valuable guide for those considering patient, participant or public engagement in the genomics context. The recognition that engagement may lead to tensions between stakeholders and the questions and considerations the framework provides on this theme, may be particularly important in the development of effective engagement strategies. Moreover, these themes and questions are likely to be of relevance for other areas of scientific activity and large-scale data initiatives. This also the case with the memorable extension of the F.A.I.R. principles to incorporate [E.]quitable and [R.]esponsible, as a proposal for ensuring that F.A.I.R. data processing results in fairer data use.

Does the article adequately reference differing views and opinions?

Yes

Are all factual statements correct, and are statements and arguments made adequately supported by citations?

Yes

Is the Open Letter written in accessible language?

Yes

Where applicable, are recommendations and next steps explained clearly for others to follow?

Yes

Is the rationale for the Open Letter provided in sufficient detail?

Yes

Reviewer Expertise:

Law, ethics and policy of genomic healthcare and research. I am not an expert on engagement but I am familiar with the discourse in relation to engagement and involvement in scientific research.

I confirm that I have read this submission and believe that I have an appropriate level of expertise to confirm that it is of an acceptable scientific standard.
